# A Comprehensive 2D-LC/MS Online Platform for Screening of Acetylcholinesterase Inhibitors

**DOI:** 10.3389/fmolb.2022.868597

**Published:** 2022-03-16

**Authors:** Claudia Seidl, Juliana Maria de Lima, Gabriel Mazzi Leme, Ananda Ferreira Pires, Dwight R. Stoll, Carmen Lúcia Cardoso

**Affiliations:** ^1^ Grupo de Cromatografia de Bioafinidade e Produtos Naturais, Departamento de Química, Faculdade de Filosofia, Ciências e Letras de Ribeirão Preto, Universidade de São Paulo, Ribeirão Preto, Brazil; ^2^ SEPARARE Núcleo de Pesquisa Em Cromatografia, Departamento de Química, Universidade Federal de São Carlos, São Carlos, Brazil; ^3^ Department of Chemistry, Gustavus Adolphus College, St. Peter, MIN, United States

**Keywords:** immobilized enzyme reactor, IMER, bioreactor, bio-guided assay, natural products

## Abstract

The continuous interest in discovering new bioactive molecules derived from natural products (NP) has stimulated the development of improved screening assays to help overcome challenges in NP-based drug discovery. Here, we describe a unique platform for the online screening of acetylcholinesterase inhibitors without the need for pre-treating the sample. In the current study, we have demonstrated the ability to combine reversed-phase separation with a capillary immobilized enzyme reactor (cIMER) in two-dimensional liquid chromatography system coupled with mass spectrometry detection. We systematically investigated the effects of method parameters that are of practical significance and are known to affect the enzyme assay and interfere in the analysis such as: bioreactor dimensions, loop sizes, amount of immobilized enzyme, second dimension flow rates, reaction time, substrate concentration, presence of organic modifier, limit of detection and signal suppression. The performance of this new platform was evaluated using a mixture containing three known AChE inhibitors (tacrine, galanthamine and donepezil) and an ethanolic extract obtained from the dry bulbs of *Hippeastrum calyptratum* (Amaryllidaceae) was investigated to provide a proof of concept of the applicability of the platform for the analysis of complex mixtures such as those derived from NPs.

## 1 Introduction

Natural products (NP) remain an important source of new chemical entities and play a key role in drug discovery. NP crude extracts are a rich source of new structurally complex compounds and offer enormous scaffold diversity (Newman and Cragg, 2016). However, the inherent complexity of NP crude extracts makes isolation and characterization of bioactive NPs time-consuming and incompatible with high-throughput bioactivity assays. Classic approaches, such as bioactivity-guided fractionation strategies followed by offline microplate-based bioassays, are effective but have significant drawbacks, especially when it comes to evaluating active compounds present at low concentrations (Cieśla and Moaddel, 2016). In addition, the high resolution obtained in the offline separation step might be lost, thereby preventing the biological effect from being correctly attributed to a specific chemical compound. In this context, developing a reliable automated screening assay for online identification and characterization of bioactive compounds in NP crude extracts is desirable (Fu et al., 2019).

In the early 1990s, direct post-column coupling of a continuous-flow enzyme assay to a liquid chromatographic (LC) system with UV-Vis, fluorescence or mass spectrometry (MS) detection received attention since it eliminated the fractionation steps (Greis, 2007; Shi et al., 2009; Malherbe et al., 2012; Zhang et al., 2013; Peng et al., 2015; De-qiang et al., 2016; Zhao et al., 2016). This post-column screening format coupled with MS detection is a relevant tool for online enzymatic activity/affinity screening and was subsequently used to identify bioactive compounds in NP crude extracts (Schenk et al., 2003; van Elswijk et al., 2003; Kool et al., 2011; Guo et al., 2018; Wu et al., 2018).

The main challenge of direct post-column coupling of a bioassay to an LC system is to overcome the solvent incompatibility between the high organic composition of the mobile phase required for an efficient separation of NP crude extracts and the physiological conditions required for the bioassay without compromising MS analysis (Kool et al., 2011). Verpoorte and collaborators were the first to develop (Ingkaninan et al., 2000a) and apply (Ingkaninan et al., 2000b) a method for coupling high-performance liquid chromatography, UV and MS detection (HPLC-UV/MS), and online post-column biochemical characterization to identify acetylcholinesterase (AChE) inhibitors in NP crude extracts. Pharmacological inhibition of cholinesterase (ChE) enzymes (acetylcholinesterase (AChE) and/or butyrylcholinesterase) is the most common treatment for Alzheimer’s disease (AD) and its associated complications (Grutzendler and Morris, 2001). Although this system was successfully applied to evaluate the AChE inhibitory effect of an alkaloid-rich fraction of *Narcissus* “Carlton,” where the known AChE inhibitor galanthamine was identified, the organic modifier in the HPLC mobile phase severely reduced the enzyme activity even though the methanol concentration was kept under 30% (Ingkaninan et al., 2000b). This work was immediately followed by others that aimed to improve sensitivity, and expand detection options for the bioassay to include fluorescence and MS (de Boer et al., 2004; de Jong et al., 2006). Nevertheless, all of these approaches still required large amounts of enzymes and other reagents (enzyme substrate and colorimetric agents) to be constantly flowed into the reaction coil, and the bioassays were heavily affected by the presence of organic modifiers.

Immobilized enzyme reactors (IMER) consisting of the desired immobilized enzyme can be easily integrated into the HPLC system to replace the traditional solution-based reaction coil and can be used to identify specific inhibitors, providing information about the mode of inhibition and allowing calculation of the inhibition constant (K_i_) using frontal (Zhang et al., 2001; de Boer et al., 2004; de Jong et al., 2006; De Moraes et al., 2014) or zonal chromatographic techniques (da Silva et al., 2013; Lima et al., 2016; Ferreira Lopes Vilela and Cardoso, 2017; Seidl et al., 2019). This approach overcomes the need for constant use of enzyme solution allowing the enzyme to be reused, while also enhancing enzyme stability in the presence of organic modifiers and pH variations (Liu et al., 2018; De Simone et al., 2019). Our group has explored different applications of immobilized capillary enzyme reactor (cIMER) as a powerful tool for online ligand screening when coupled with UV (da Silva et al., 2013; Vilela et al., 2014; Lima et al., 2016; Magalhães et al., 2016) and MS detection (Vanzolini et al., 2013; Ferreira Lopes Vilela and Cardoso, 2017; Seidl et al., 2019). More recently, Yuan et al. (Yuan et al., 2020) developed a method that coupled LC separation with MS detection and an immobilized enzyme reactor (HPLC-IMER-MS) for screening of AChE inhibitors in NP crude extracts. They successfully applied the method to the extract of a natural plant, *Lycoris radiata*, which is known to contain AChE inhibitors such as galanthamine and lycoramine and were able to identify a novel AChE inhibitor (dihydro-latifaliumin C) (Yuan et al., 2020).

Our main objective was to develop a fully automated and easy to assemble online platform for screening AChE inhibitors without the need for any sample pretreatment. We used a commercially available two-dimensional liquid chromatography system coupled with MS detection (2D-LC-MS) where the sample was separated in the first dimension (^1^D), while the bioassay with MS-detection occurred in the second dimension (^2^D). An 8-port/2-position high pressure switching valve equipped with two identical sample loops was used for the time-based fraction transfer of the effluent from the ^1^D column to the second dimension where each fraction (combined with the enzyme substrate) was then screened for its potential to inhibit the acetylcholinesterase enzyme. We systematically investigated the effects of method parameters that are of practical significance and are known to affect the enzyme assay and interfere with the analysis. These parameters include bioreactor dimensions, loop sizes, amount of immobilized enzyme, reaction time, substrate concentration, and presence of organic modifier. A mixture containing three known AChE inhibitors (tacrine, galanthamine and donepezil) was used during the development and validation of the platform. Finally, an ethanolic extract obtained from the dry bulbs of *Hippeastrum calyptratum* (Amaryllidaceae) was investigated to provide a proof of concept of the applicability of the platform for the analysis of complex mixtures such as those derived from NPs.

## 2 Materials and Methods

### 2.1 Chemical and Reagents

Acetylcholinesterase from *Electrophorus electricus* (Type VI-S; EC 3.1.1.7) (eelAChE), acetylcholine iodide (≥97%, ACh), choline chloride (≥99%, Ch), galanthamine hydrobromide from *Lycoris* sp (≥94% HPLC), 9-amino-1,2,3,4-tetrahydroacridine hydrochloride hydrate (tacrine hydrochloride, ≥99%), donepezil hydrochloride (≥98% HPLC), edrophonium chloride, caffeine, ammonium acetate, Tris hydrochloride, glutaraldehyde (25% aqueous solution), and sodium phosphate monobasic were purchased from Sigma-Aldrich (St. Louis, United States). 3-(Aminopropyl)trietoxysilane (≥99%, APTES) was purchased from Acros Organics (Geel, BE). HPLC grade methanol (MeOH), acetonitrile (ACN) and ethanol (EtOH) were acquired from Merck-Millipore (Burlington, United States). Ultrapure water was obtained with a Merck-Millipore Milli-Q^®^ system (Burlington, United States). Fused silica capillary (0.375 mm o. d. x 0.1 mm i. d.) was acquired from Polymicro Technologies (Phoenix, United States). All the solutions were filtered through a Merck-Millipore nylon membrane filter (0.22 μm) prior to use (Burlington, United States).

### 2.2 Hippeastrum calyptratum Methanolic Extract

Bulbs of the species *Hippeastrum calyptratum* (Amaryllidaceae) were collected in the city of Cunha (São Paulo, Brazil), identified by Mr. Mauro Peixoto and Dr. Jullie Dutilh (University of Campinas, Unicamp, Brazil) and a voucher deposited at the Herbarium of the Instituto plantarum (HPL 13043). An ethanolic crude extract was obtained by cold maceration until exhaustion, evaporated under reduced pressure and stored in a freezer. The sample was (10 mg ml^−1^) resuspended in ethanol and centrifuged at 10,000 rpm for 5 min before performing the analyses. Access to genetic heritage was registered at Sistema Nacional de Gestão do Patrimônio Genético e do Conhecimento Tradicional Associado (SisGen) under code ABE20A2.

### 2.3 Instrumentation and Software

The instrument consisted of modules from the 1,290 Infinity line (Agilent Technologies, Santa Clara, United States): an autosampler (G4226A), a thermostatted column compartment (G1316C), a binary pump (G4220A, referred to as *enzyme buffer pump*), a high-speed pump (G7120A), a diode-array UV detector (DAD) (G4212A, with 1.0 μL flow cell, G4212-60008), a 8-port/2-position high-pressure switching valve (Agilent G4236A, Duo Valve) equipped with two identical sample loops (Agilent 5,067–5,440, calibrated loop kit for 2D-LC) and an additional pump from the Prominence series (model LC-20AD, Shimadzu, Tokyo, Japan). The system was controlled by the 2D-LC acquisition software (A.01.04 [017]) from Agilent and the Prominence pump was manually activated. The LC system was coupled to a Bruker Amazon Speed ion trap mass spectrometer (IT-MS) equipped with an electrospray source, controlled by the Bruker Compass Hystar software (version 4.5) (Bruker Daltonics Inc., Billerica, United States). Data were acquired and analyzed with the Compass DataAnalysis software (version 4.3). MS parameters were configured as follows: capillary voltage = 5000 V; end-plate voltage = 550 V, drying gas flow rate = 11.0 L min^−1^, drying temperature = 350°C, and nebulizer pressure = 60 psi. Analyses were conducted in the manual MS(n) mode with positive ionization (*m*/*z* from 40 to 1,500) and isolation width was set at ± *m*/*z* 0.5. A syringe pump (Model 11 Plus, Harvard Apparatus, Holliston, United States) was also used for different purposes, described in the following sections.

### 2.4 AChE-cIMER Preparation

AChE immobilization was carried out by infusing the enzyme into a fused silica capillary using a syringe pump. A capillary (1,000 mm × 0.1 mm i. d.) previously activated with APTES and modified with glutaraldehyde was used as support for enzyme immobilization. The immobilization procedure was performed according to previously reported protocols (Vanzolini et al., 2013) using a 50 U mL^−1^ AChE solution. When not in use the reactor was maintained at 4°C, with both extremities immersed in Tris buffer 50 mM, pH 8. A negative control capillary (referred to as cIMER-blank) was prepared by following the same immobilization procedure except for addition of the AChE solution.

### 2.5 AChE-cIMER Activity Assay

**FIGURE 1:**
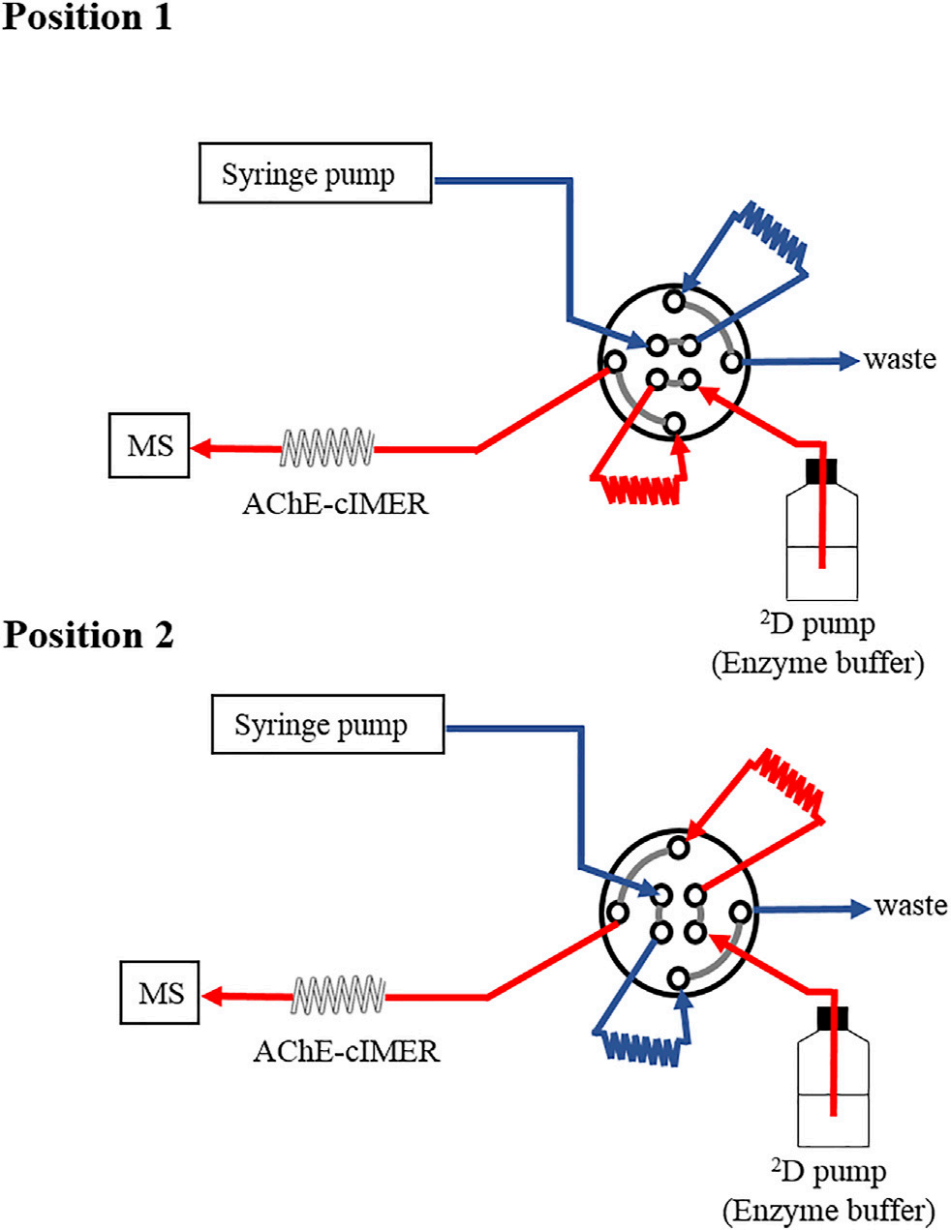
A schematic illustration of the instrument configuration used to evaluate the AChE-cIMER activity and to optimize parameters.

The AChE-cIMER catalytic activity was determined by measuring the formation of the product of ACh enzymatic hydrolysis by AChE, which is choline (detected by MS as [M + H]^+^, *m*/*z* 104.0). To this end, a simplified system configuration as illustrated in Figure 1 was used, which includes a syringe pump, a switching valve equipped with two identical sample loops, the AChE-cIMER (100 cm × 0.375 mm x 0.1 mm i. d.), the enzyme buffer pump, and the IT-MS instrument.

The syringe pump was used to fill the loops with the enzyme substrate (ACh solution prepared in 15 mM ammonium acetate solution pH 5, at 100 μl min^−1^). The valve was regularly activated (referred to as *modulation time*), connecting the flow path between the enzyme buffer pump and the previously filled loop, delivering the substrate to the AChE-cIMER in ammonium acetate solution (15 mM, pH 8), followed by MS detection.

#### 2.5.1 Optimization of AChE-cIMER Reaction Conditions

In order to achieve the proper AChE-cIMER activity, parameters such as residence time (1.0, 0.8, 0.6 and 0.3 min), loop size (20, 40, 60, and 80 µL), and enzyme buffer pump flow rate (0.4, 0.5 and 0.6 ml min^−1^) were evaluated. The effects of the type and percentage of organic modifiers as part of the substrate solution (ACN, EtOH, or MeOH) on the enzymatic activity were also studied and determined using this configuration. The syringe pump flow was set to overfill (200%) the employed loop (according to their volume and reaction time). The modulation process was repeated five times to evaluate repeatability.

#### 2.5.2 Kinetic Studies

The Michaelis–Menten saturation curve for the AChE-cIMER was obtained by using the system configuration described in Figure 1 and the following final conditions: Enzyme buffer flow rate, 600 μl min^−1^; Two sample loops, 60 μL; Modulation time, 36 s. Eight ACh solutions with concentrations in the range of 2.5–6.25 × 10^–5^ mM prepared in 15 mM ammonium acetate solution pH 5 were infused into the system using the syringe pump at 100 μL min^−1^. K_M,app_ value was obtained by using the GrapPad Prism 5.0 enzyme kinetics tool.

### 2.6 Offline AChE-cIMER Inhibition Assay

**FIGURE 2:**
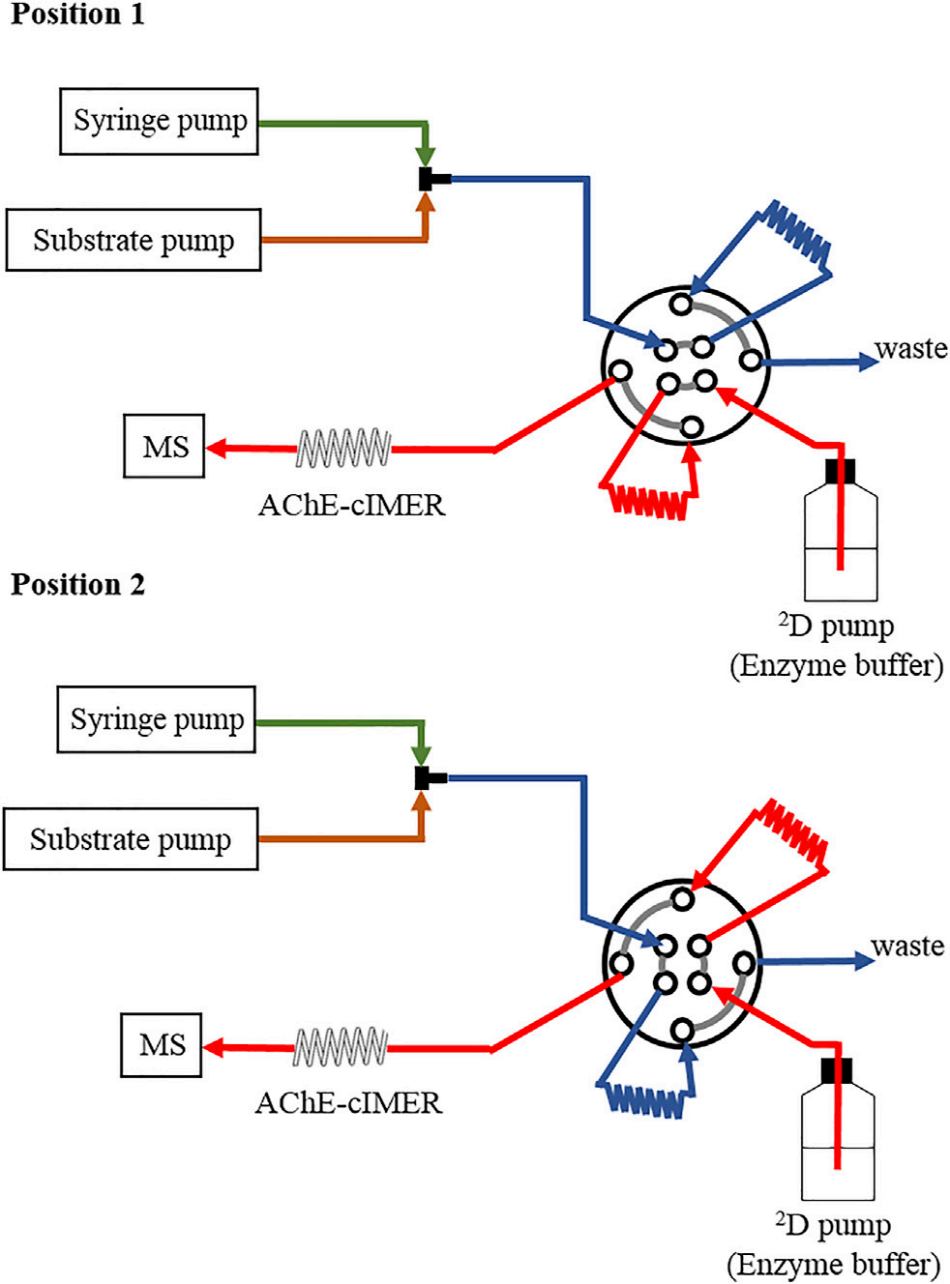
A schematic illustration of the instrument configuration used for the AChE-cIMER inhibition assay.

The offline AChE-cIMER inhibition assay was performed using the configuration shown in Figure 2. An additional pump was necessary to deliver the enzyme substrate (140 μM ACh solution, prepared in 15 mM ammonium acetate at pH 5) at 50 μL min^−1^. The syringe pump was used to deliver individual solutions of acetylcholinesterase inhibitors in different concentration ranges: galantamine (0.27–27 μM); tacrine (4.2 × 10^−3^–4.2 μM) and donepezil (2.3 × 10^–3^ to 2.3 μM), which were all prepared in 15 mM ammonium acetate solution at pH 8. The syringe pump flow was 50 μL min^−1^. Substrate and inhibitor solutions were combined through a T-piece placed before the switching valve (Figure 2), which resulted in a final flow rate of 100 μL min^−1^ reaching the sample valve. The valve was equipped with two 60 μL sample loops and switched every 36 s (modulation time = 36 s), to deliver 60 μL of the substrate + inhibitor mixture to the IMER at each modulation. In Position 1, the substrate + inhibitor solution was stored in *loop 1,* whereas the enzyme buffer pump (15 mM ammonium acetate pH 8.0, at 600 μL min^−1^) conditioned the AChE-cIMER and kept the MS baseline steady. In Position 2, the content of *loop 1* was directed to the AChE-cIMER for analysis, while *loop 2* was filled with more substrate + inhibitor solution. Total analysis time was 10 min. A negative control experiment was performed by replacing the cIMER with the cIMER-blank.

The inhibition percentage for each inhibitor concentration was calculated using Eq. 1 and peak areas from extracted ion chromatograms for the molecular ion of protonated choline (Ch, *m/z* 104.0):

$$inhibition\%=\left[1-\frac{A_{i}-C_{i}}{A_{0}-C_{0}}\right]x100$$ (1)

where A_*i*_ and C_*1*_ are the areas obtained in the presence of inhibitors with the AChE-cIMER and cIMER-blank, respectively, and A_*0*_ and C_*0*_ are the areas obtained in the absence of inhibitors with the AChE-cIMER and cIMER-blank, respectively. All samples were analyzed in triplicate and results are presented as means ± one standard deviation (GraphPad Prism version 5.0).

### 2.7 Online 2D-LC-MS Platform for AChE-cIMER Inhibition Assay

**FIGURE 3:**
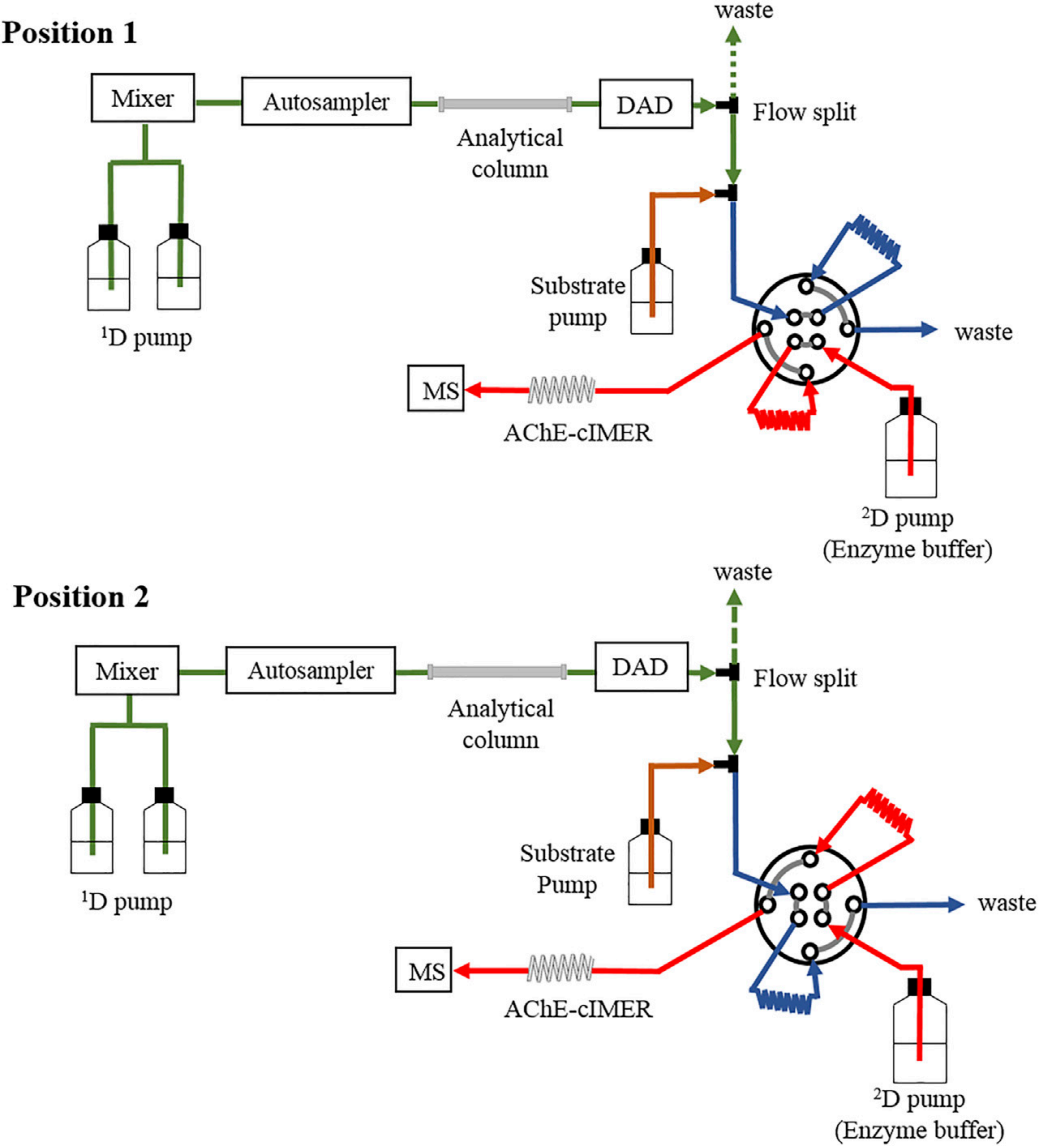
A schematic illustration of the instrument configuration used for the complete online 2D-LC-MS platform using the AChE-cIMER screening assay.

The 2D-LC-MS system was configured with the previously described modules (see *Instrumentation and software* section) as illustrated in Figure 3. The first dimension (^1^D) included a binary pump, autosampler, thermostatted column compartment, a Gemini C18 analytical column (150 mm × 1.0 mm i. d., 5 μm, 110 Å, Phenomenex, Torrance, United States) and diode array detector. The second dimension (^2^D) consisted of the enzyme buffer pump, the Prominence pump (added to the system for exclusive delivery of enzyme substrate solution), and the immobilized acetylcholinesterase enzyme reactor (1,000 mm × 0.1 mm i. d.), which was finally coupled to the ion trap mass spectrometer instrument (IT-MS). Both dimensions were interfaced with an 8-port/2-position high-pressure switching valve equipped with two 60-µL sampling loops.

Samples were analyzed using the online 2D-LC-MS platform for AChE-cIMER inhibition assay according to the following procedure:

When the valve was in position 1, sample was injected (10 μL) into a Gemini C18 analytical column (150 mm × 1.0 mm i. d., 5 μm, 110 Å, Phenomenex, Torrance, United States) held at 40°C. The binary mobile phase consisted of (A) 20 mM ammonium acetate at pH 6 and (B) methanol. A solvent gradient was employed from 5-100-100% B in 0-10-12 min for the known inhibitors and 0-40-42 min for the *H. calyptratum* crude extract with equilibration time of 5 min (post-run time). The flow rate was 100 μL min^−1^. The RP-LC effluent was split 1:1 (1 part to waste and 1 part to the switching valve) and combined with the substrate solution (ACh, 140 μM in 15 mM ammonium acetate at pH 5) at 50 μL min^−1^ before the switching valve by means of a T-piece (total flow of 100 μL min^−1^ reaching the valve).

While the first fraction combined with the enzyme substrate fills the first sample loop (60 μL) the ^2^D pump (enzyme buffer pump) delivering the enzyme buffer (15 mM ammonium acetate at pH 8) at 600 μL min^−1^ runs through the second loop (60 μL) keeping the AChE-cIMER conditioned and MS base line steady. After 36 s (modulation time) the valve switches position and, when in position 2, the content of the first loop (fraction + substrate solution) is flushed out by the enzyme buffer pump (15 mM ammonium acetate at pH 8) at 600 μL min^−1^ towards the AChE-cIMER for MS analysis while the second loop is filled with the next following fraction eluting from the analytical column + substrate solution. Total analysis time was 10 min.

#### 2.7.1 Assay Selectivity and Specificity

To assess the assay selectivity, 10 μL of individual solutions and a final mixture containing the same known AChE inhibitors were injected into the system. Galanthamine, tacrine, and donepezil were prepared at 108, 8.4, and 6 μM, respectively (in 15 mM ammonium acetate at pH 8). Assay specificity was evaluated by injecting 10 μL of a solution containing caffeine at 400 μM prepared in 15 mM ammonium acetate at pH 8.

#### 2.7.2 Ion Suppression Evaluation

To evaluate a possible suppression of the signal corresponding to the product of the enzymatic reaction choline (Ch ion, *m*/*z* 104.0) by the known inhibitors, the AChE-cIMER was replaced by the cIMER-blank (no enzyme present). A solution of choline at 10 μM (15 mM ammonium acetate at pH 5) was used instead of the substrate solution and the peak area corresponding to the Ch ion was compared with the corresponding peak area obtained after 165, 15, and 25 μM (prepared in 15 mM ammonium acetate pH 8) of galantamine, tacrine, and donepezil respectively were injected into the system.

## 3 Results

### 3.1 AChE-cIMER Activity and Optimization

**FIGURE 4:**
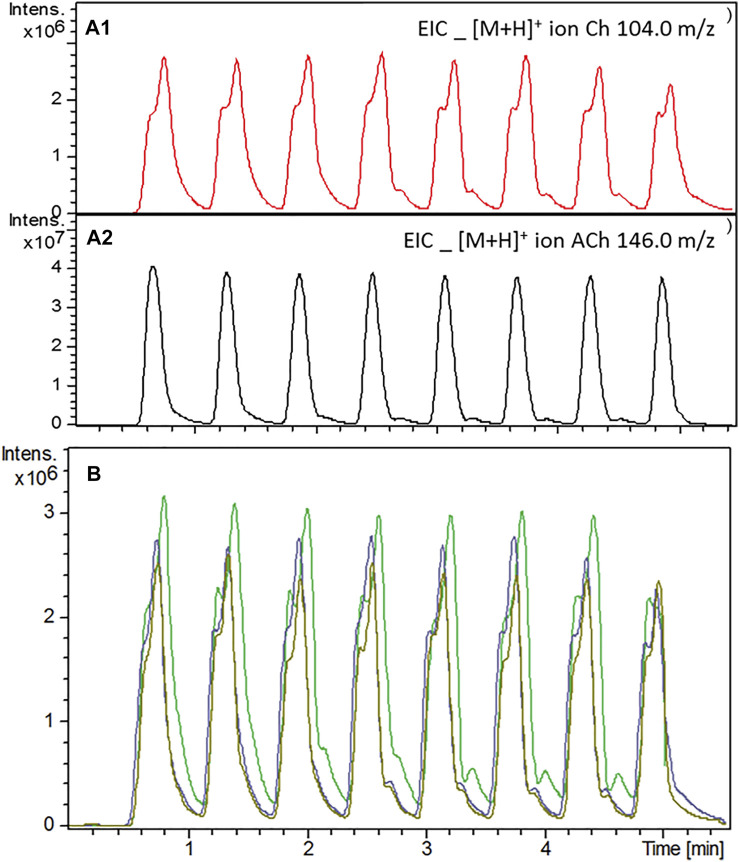
AChE activity evaluation after the immobilization procedure using the system configuration shown in Figure 1. **(A1)** Extracted ion chromatogram (EIC) for the protonated molecular ion of Ch (*m/z* 104.0) obtained when a solution of 70 μM ACh was constantly infused into the system at 100 ml min^−1^. **(A2)** EIC of excess unreacted ACh (molecular ion for protonated Ach; *m/z* 146.0) after enzymatic hydrolysis. Assay conditions are described in the experimental section. **(B)** Effect of the enzyme buffer pump (15 mM ammonium acetate pH 8.0) (Ch ion, *m/z* 104.0) (--) 0.4 ml min^−1^, (--) 0.5 ml min^−1^ and (--) 0.6 ml min^−1^flow rate on the AChE-cIMER activity.

Acetylcholinesterase activity was investigated after the immobilization procedure using the system configuration illustrated in Figure 1. The immobilization procedure did not affect the enzymatic activity and the amount of enzyme used in the immobilization procedure (50 U mL^−1^) proved to be enough to deliver a reliable, reproducible, and detectable signal for the protonated molecular ion of choline (*m/z* 104.0), the product of the enzymatic reaction (Figure 4). It was possible to monitor AChE-cIMER activity even with substrate concentration as low as 6.25 × 10^−5^ μM (Supplementary Figure S1A). Spontaneous hydrolysis of the substrate was not detectable—only noise signal corresponding to the Ch product ion (*m/z* 104.0) was detected when the substrate solution was infused through the cIMER-blank (Supplementary Figure S2). As expected, the presence of ACN and EtOH at concentrations as low as 20% caused the enzyme to completely lose its catalytic activity (data not shown), while MeOH was well tolerated at concentrations up to 50%. However, at 50% MeOH a small loss of enzymatic activity was observed as a decrease in signal intensity for the choline ion. Nevertheless, the enzymatic activity was recovered and remained reproducible with each new injection. The enzymatic reaction proved to be fast and reproducible, even with modulation times as low as 0.3 min (18 s) for all the ^2^D flow rates tested. However, on the basis of peak shape, the following were chosen as final conditions: modulation time of 36 s, a 60 μL sample loop, and ^2^D flow rate of 600 μl min^−1^.

The AChE-cIMER stability was evaluated over time according to its ability to maintain its catalytic activity by monitoring the choline signal corresponding to the enzymatic reaction product (Ch, *m/z* 104.0) using the configuration described in Figure 3 and the optimized final conditions. After 3 months of daily use, the Ch signal did not decrease, remaining visible with an intensity above x10^6^ and was still active 2 years after the immobilization.

#### 3.1.1 Kinetic Studies

Kinetic studies were performed using the system configuration described in Figure 1. Eight different ACh solutions with concentrations ranging from 2.5 to 6.25 × 10^–5^ mM (three replicates for each concentration) were infused one at the time at 100 μL min^−1^ and 60 μL fractions (sample loop size) were transferred to the AChE-cIMER every 36 s while the enzyme buffer pump flow rate was set at 600 μl min^−1^ (Supplementary Figure S1B).

From the Michaelis–Menten saturation curve for the AChE-cIMER we obtained K_M,app_ (64.39 ± 6.58 μM). To ensure that appropriate conditions were used for inhibitors with different mechanisms, AChE-cIMER inhibition screening assays were performed at substrate concentrations close to K_M,app_ value (70 µM) (Copeland, 2016).

### 3.2 Offline AChE-cIMER Inhibition Assay

The offline AChE-cIMER inhibition assay was performed using the system configuration described in Figure 2. Briefly, solutions containing standard AChE inhibitors tacrine, donepezil and galanthamine at least five different concentrations were infused at 50 μL min^−1^ and combined with the substrate solution that was being pumped at 50 μL min^−1^. The final solution containing inhibitor + substrate was transferred to the AChE-cIMER every 36 s while the enzyme buffer pump flow rate was set at 600 μl min^−1^.

**FIGURE 5:**
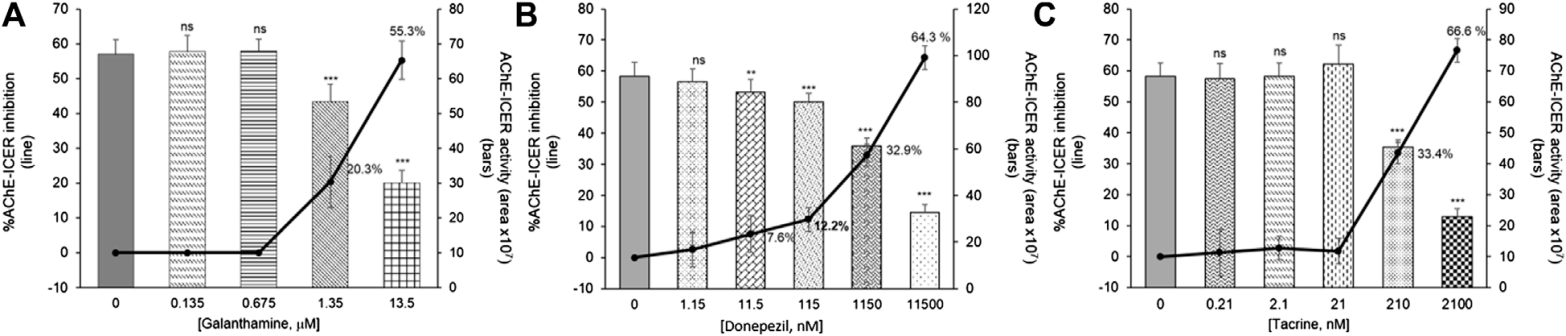
Effect of different concentrations of standard inhibitors (galanthamine, donepezil and tacrine) on the AChE-cIMER activity (bars) and individual inhibition percentages (solid lines) obtained using the system configuration shown in Figure 2. Conditions: Syringe pump flow rate, 50 mL min^−1^; Substrate pump flow rate of 50 mL min^−1^ containing 140 mM ACh solution prepared in 15 mM ammonium acetate at pH 5; Sample loop, 60 ml; Modulation time, 36 s; Enzyme buffer pump flow rate (delivering the substrate + inhibitor solution to the ICER in 15 mM ammonium acetate at pH 8), 600 mL min^−1^. Results expressed as means ± one standard deviation from 12 replicates (ns = *p* > 0.05; ^**^ *p* ≤ 0.01; ^***^ *p* ≤ 0.001).

Enzyme inhibition was visible at inhibitor concentrations as low as 1.35 μM for galanthamine (20.3% inhibition), 1.15 μM for donepezil (32.9% inhibition), and 0.21 μM for tacrine (33.4% inhibition). Meanwhile, inhibitor solutions with 10-fold higher concentrations resulted in 55.3, 64.3 and 66.6% enzyme inhibition for galanthamine, tacrine, and donepezil, respectively (Figure 5). The concentration of each inhibitor that caused an enzyme inhibition above 50% was used as a reference to prepare individual inhibitor solutions and a solution containing a mixture of the three aforementioned inhibitors for the online 2D-LC-MS platform for AChE-cIMER inhibition assay.

### 3.3 Online 2D-LC-MS Platform for AChE-cIMER Assay

First, we evaluated the ability of the online 2D-LC-MS platform for AChE-cIMER inhibition screening assay to individually detect the inhibition potential for the same known inhibitors, galanthamine, tacrine, and donepezil at 108, 8.4 and 6 μM (all in 15 mM ammonium acetate at pH 8), respectively. Each inhibitor solution was injected (10 μL) into the analytical column and then eluted using the chromatographic conditions described in the experimental section. Half of the eluate was combined with the enzyme substrate solution (140 μM in 15 mM ammonium acetate pH 5) and half went to the trash. Every 36 s, a 60-μL aliquot was transferred to the second dimension toward the AChE-cIMER, and the enzyme inhibition was measured.

**FIGURE 6:**
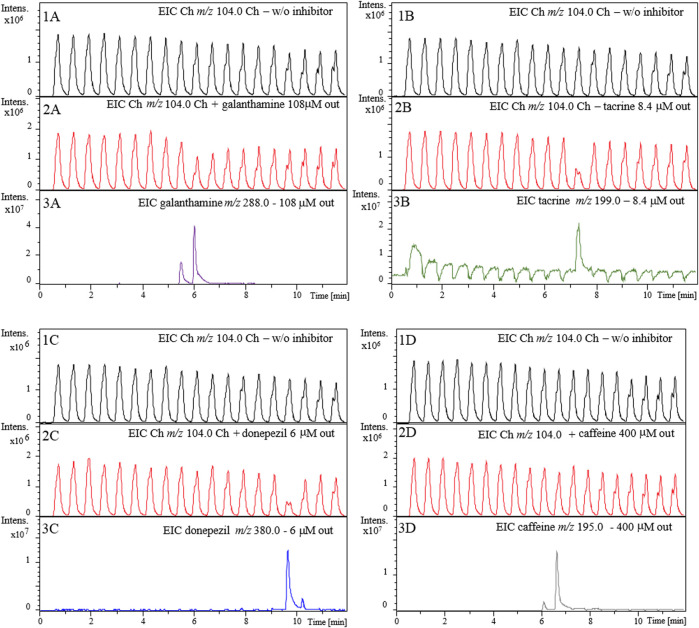
Extracted ion chromatograms (EICs) for the enzyme reaction produce choline (*m/z* 104.0) in the absence (Panel **1A-1D**) and presence of 108 μM galanthamine **(2A)** 8.4 μM tacrine, **(2B)**, 6 μM donepezil **(2C)** and 400 μM caffeine **(2D)**. Panels **3A-3D** show EICs from 1D elution of galanthamine (*m/z* 288.0), tacrine (*m/z* 199.0), donepezil (*m/z* 380.0), and caffeine (*m/z* 195.0), respectively. 1D RP-LC chromatographic conditions: 5-100-100% B in 0-10^−12^ min with equilibration time of 5 min (post-run time). The flow rate was 100 μl.min^−1^ with injection volume of 10 ml. Mobile phase: **(A)** 20 mM ammonium acetate pH 6 and **(B)** methanol. Substrate pump (ACh 140 mM in 15 mM ammonium acetate pH 5) at 50 ml.min**−1**. 2D pump (15 mM ammonium acetate pH 8) flow rate was fixed at 600 ml.min^−1^. The modulation time was 36 s.

Figure 6 shows the resulting extracted ion chromatogram (EIC) for the protonated molecular ion choline (Ch, *m/z* 104.0) after the injection of individual solutions of galanthamine (Figure 6 panel 2A), tacrine (Figure 6 panel 2B) and donepezil (Figure 6 panel 2C). It is possible to observe that the choline signal intensity (Panels 2A, 2B and 2C) decreased when compared to the control (analysis performed in the absence of inhibitor; Panels 1A, 1B and 1C) and this decrease corresponds to the exact modulation where each inhibitor was present. In previous works the enzymatic activity was strongly impaired in the presence of the organic modifier, although the MeOH concentration was kept as low as 25% (Ingkaninan et al., 2000b; Yuan et al., 2020). Here, the enzymatic activity was only slightly affected when concentration of MeOH in the ^1^D effluent reached 100% (Figure 6 Panel 1A-D), and the enzyme activity was recovered prior to the next injection during flushing with 15 mM ammonium acetate solution at pH 8.

The signal intensity of the enzymatic reaction product decreased (*m/z* 104.0) (Panels 2A, 2B and 2C) when compared to the control (obtained in the absence of inhibitor; Panels 1A, 1B and 1C) at the exact retention time corresponding to the modulations where the inhibitors were present (Panels 3A, 3B and 3C). The peak corresponding to galanthamine when transferred from the analytical column to the AChE-cIMER was divided into two modulations. Panel 3A shows the EIC corresponding to galanthamine (*m/z* 288.0) at about 6 min. In panel 2A, it is possible to observe a decrease in the signal intensity of the Ch (*m/z* 104.0) when compared to the control (Panel 1A) around the same retention time matching the modulations where galanthamine was present, thus proving the occurrence of enzymatic inhibition. The same reasoning can be used to analyze the results for tacrine and donepezil. The EIC corresponding to tacrine (*m/z* 199.9) and donepezil (*m/z* 380.0) can be observed close to 7.5 and 10 min, respectively and modulations corresponding to these retention times show a decrease in Ch (*m/z* 104.0) signal intensity. Caffeine was used to assess the assay’s specificity for true AChE inhibitors. As shown in panel 2D, caffeine (*m/z* 195.0) did not inhibit AChE even when tested at a high concentration of 400 µM. These results confirmed the sensitivity and specificity of the 2D-LC-MS online platform for the AChE-cIMER inhibition screening assay.

**FIGURE 7:**
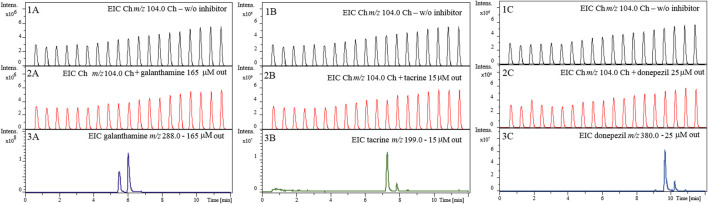
Evaluation of choline ion suppression. Extracted ion chromatograms (EICs) for choline (*m/z* 104.0) in the presence of 165 µM galanthamine (2A) 15 µM tacrine (2B) and 25 µM donepezil (2C). EICs for galanthamine (*m/z* 288.0), tacrine (*m/z* 199.0), and donepezil (*m/z* 380.0) are shown in Panels 3A–3C, respectively. Chromatographic conditions are the same as the one described in Figure 6.

Next, we evaluated whether the inhibition observed for the known inhibitors in the previous experiment (Figure 6) was a result of true inhibition or just a signal suppression effect. As shown in Figure 7, we observed no effect on the signal corresponding to the Ch ion (*m/z* 104.0) in the absence (Figure 7, Panels 1A–1C) and presence (Figure 7, Panels 2A–2C) of known inhibitors, even when tested at concentrations much higher than those used for the inhibition assay shown in Figure 6. This confirms that the observed decreases in peak intensity for the Ch ion (*m/z* 104.0) (Figure 6) were actually due to interaction between each of the known inhibitors and the AChE immobilized inside the enzyme reactor and therefore true enzyme inhibition.

**FIGURE 8:**
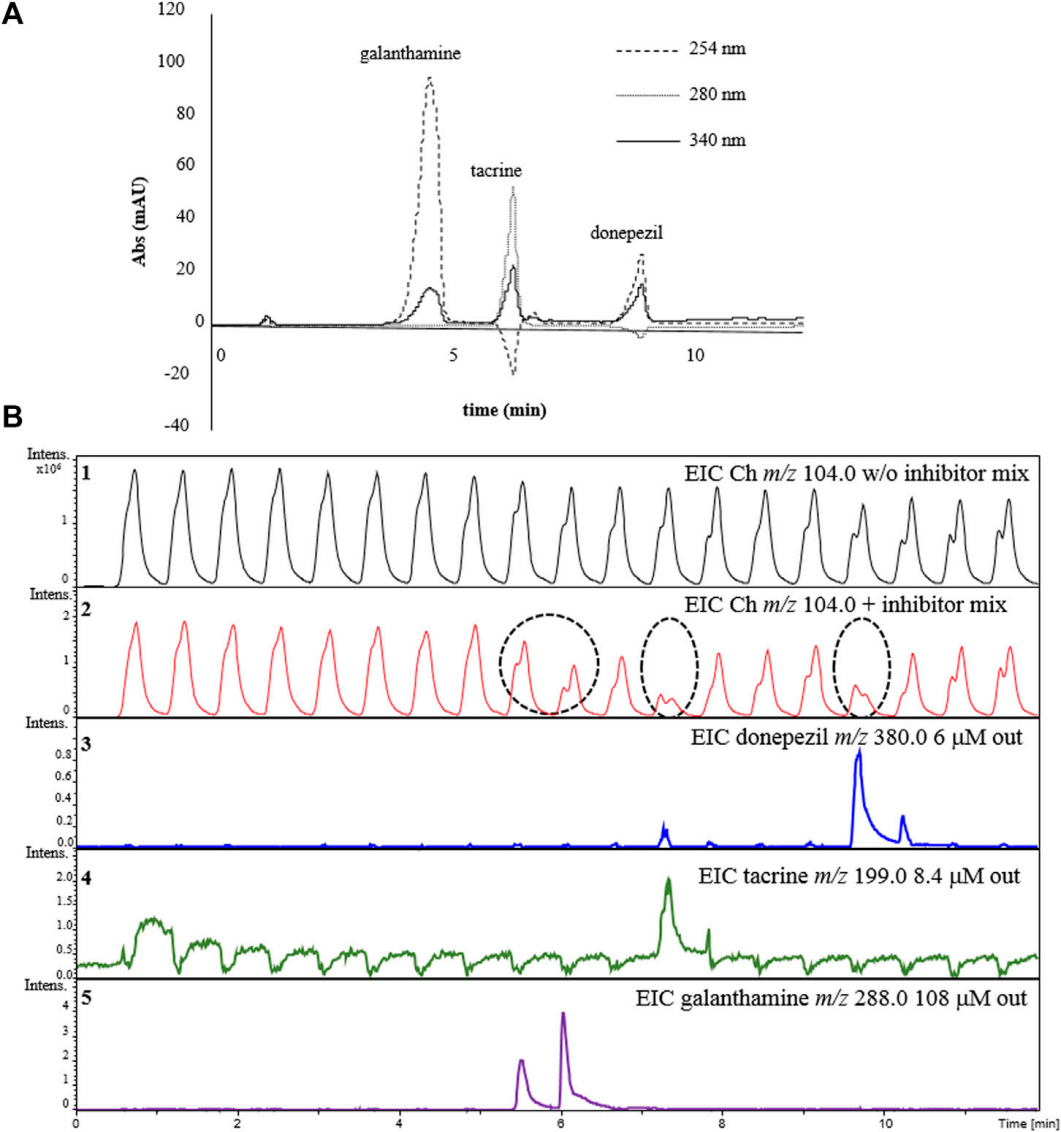
Demonstration of the detection of AChE inhibitors in a mixture using the online 2D-LC-MS. **(A)** ^1^D RP-LC separation of the inhibitor mixture and **(B)** extracted ion chromatograms (EICs) for choline (*m/z* 104.0) in the absence (Panel 1) or presence of the inhibitor mix (Panel 2). Panels 3-5 show the EICs for each individual inhibitor in the mix: donepezil *m/z* 380.0 (Panel 3); tacrine (*m/z* 199.0) (Panel 4) and galanthamine *m/z* 288.0 (Panel 5). Dashed circles indicate the modulations where the inhibition occurred and the dashed line relates them to the elution time of each respective inhibitor from the ^1^D separation. Chromatographic conditions are the same as the one described in Figure 6.

A mixture containing all the three inhibitors at the same concentrations tested individually was prepared and injected it into the complete 2D-LC-MS online platform for the AChE-cIMER inhibition screening assay. RP-LC retention times for galanthamine, tacrine, and donepezil were 4.3, 5.9 and 8.5 min, respectively (Figure 8A). The enzyme inhibition profile resulting from the analysis of the inhibitor mixture was similar to the inhibition profile obtained for each inhibitor when analyzed individually. To analyze the data shown in Figure 8B, we compared the EIC signal obtained for the Ch ion (*m/z* 104.0) in the absence (Panel 1) and presence (Panel 2) of the known inhibitor mixture. Panel 2 shows a decrease in signal intensity corresponding to the enzymatic reaction product (*m/z* 104.0) matching the modulations where the known inhibitors were present. Galanthamine was the first inhibitor to elute and once again its peak was divided (not equally) between two modulations (Figure 8B panel 5). Therefore, it is possible to confirm a decrease in the intensity of the Ch signal close to 5.5 and 6 min corresponding to the modulations where galanthamine was present (Figure 8B Panel 2). Following the same reasoning, tacrine (Figure 8B Panel 4) and donepezil (Figure 8B Panel 3) were the second and third compounds to elute and their respective inhibitory effects could be verified by a decrease in the intensity of the signal corresponding to the product of the enzymatic reaction around 7.5 and 10 min coinciding with the modulations where they were present (Figure 8B Panel 2).

**FIGURE 9:**
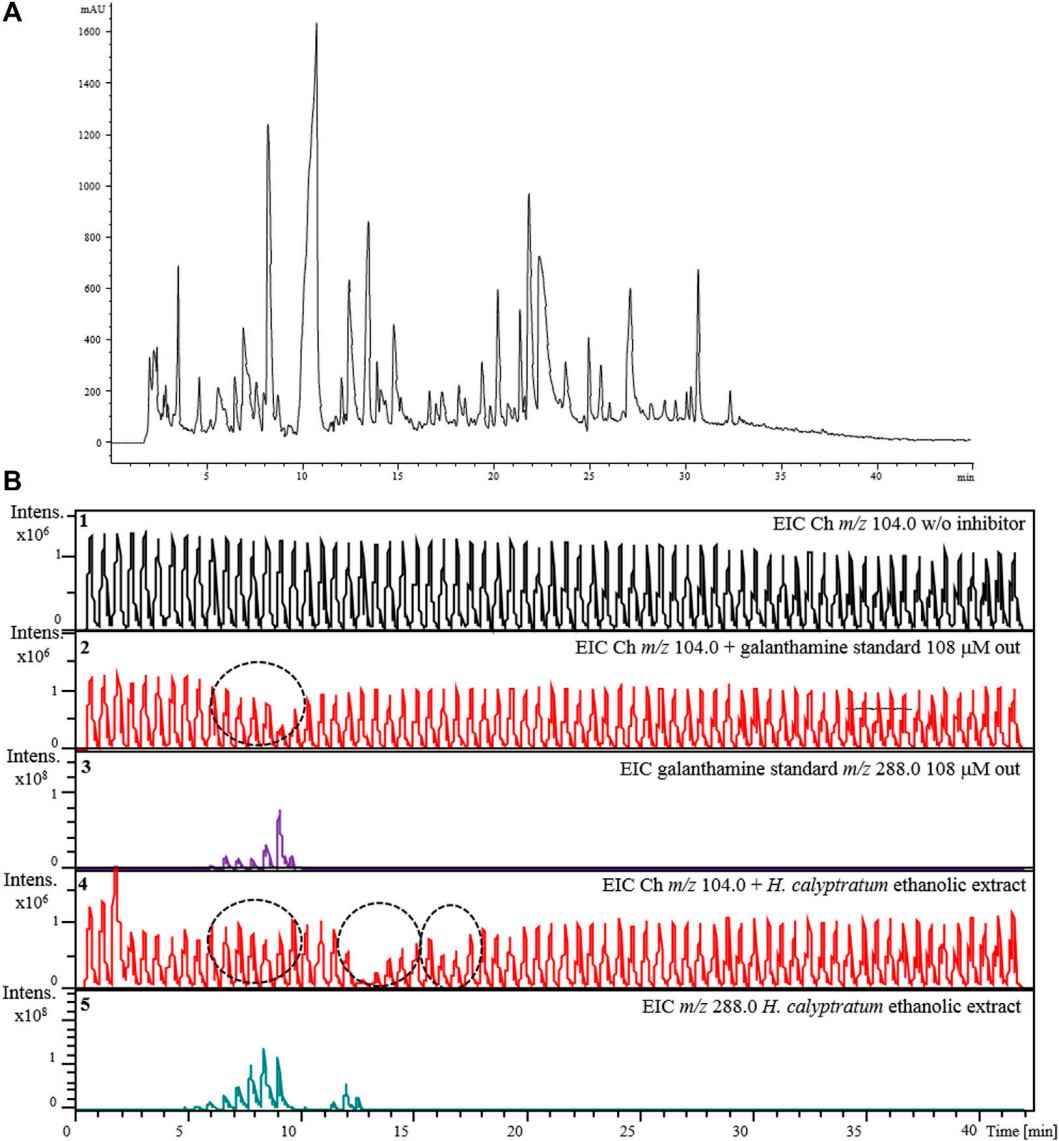
Demonstration of the applicability of the complete 2D-LC-MS online platform for the AChE-cIMER inhibition screening assay. **(A)** ^1^D RP-LC separation of the *H. calyptratum* ethanolic extract (10 mg ml^−1^) and **(B)** extracted ion chromatograms (EICs) for choline (*m/z* 104.0) in the absence (Panel 1), presence of the standard galanthamine solution (108 uM) (Panel 2) or ethanolic extract solution (Panel 4). Panel 3 and 5 corresponds to the EICs (*m/z* 288.0) for galanthamine when injected alone and present within the *H. calyptratum* ethanolic extract, respectively. Dashed circles indicate the modulations where the inhibition occurred. ^1^D RP-LC chromatographic conditions: 5–100–100% B in 0–40–42 min with equilibration time of 5 min (post-run time). The flow rate was 100 μL min^−1^ with injection volume of 10 μL. **(A)** 20 mM ammonium acetate pH 6 and **(B)** methanol. Substrate pump (ACh 140 μM in 15 mM ammonium acetate pH 5) at 50 μL min^−1^. ^2^D pump (15 mM ammonium acetate pH 8) flow rate was fixed at 600 μL min^−1^. The modulation time was 36 s.

Lastly, an ethanolic extract solution (10 mg ml^−1^) obtained from bulbs of *H. calyptratum* was used to verify the applicability of the assay using the complete 2D-LC-MS online platform (Figure 3). Figure 9A show the chromatographic profile after injection of the *H. calyptratum* extract while the results for the AChE inhibitor screening assay are shown in Figure 9B. Experimental details are given in Section 2.7. Figure 9B shows the EIC signal corresponding to the product of the enzyme reaction *m/z* 104.0 in the absence of any inhibitor (Panel 1), after the injection of the inhibitor galanthamine (GL) solution (108 μM) (Panel 2) and after the injection of the *H. calyptratum* ethanolic extract (Panel 4). Panel 3 and 5 corresponds to the EICs (*m/z* 288.0) for galanthamine when injected alone and present within the *H. calyptratum* ethanolic extract, respectively. It is possible to observe 3 regions (Figure 9, panel 4) where the extract showed a possible anticholinesterase activity. The first, with less intense inhibition corresponding to modulations between 5 and 10 min, the second region with a more intense and visible inhibition between 11 and 14 min and, a third region with inhibition of intermediate intensity visible between 16 and 18 min. The first region coincides with modulations and *m/z* corresponding to galanthamine used as a control (*m/z* 288.0), while the second and third may indicate one or a set of compounds with greater anticholinesterase activity within the extract. More in-depth studies should be carried out to try to identify those responsible for this activity present in the extract and are not part of the scope of this work.

## 4 Discussion

Natural products (NP) have an incalculable potential as key structures for the discovery of new drugs due to their high chemical diversity, biochemical specificity and other molecular properties. However, the incompatibility of crude extract with high-throughput assay procedures compromises the success of the natural products approach in the drug discovery process (Newman and Cragg, 2016). Therefore, to overcome the technical barriers to screening for bioactivity in natural products, it is necessary to develop improved automated, fast and robust analytical methodologies. The objective of the work was the development of a complete automated online screening platform to evaluate the AChE inhibition potential of complex mixtures such as those derived from NPs without the need for pre-treating the sample.

The 2D-LC system configuration allows an easy combination of two completely incompatible types of analysis into a single method. Thus, the sample was separated in the first dimension (^1^D) using RP-LC in gradient mode, while the aqueous bioassay with MS detection took place in the second dimension (^2^D). An 8-port/2-position high pressure switching valve equipped with two identical sample loops was used as the system interface. The main advantage of using a switching valve as a system interface instead of using direct post-column coupling of a continuous-flow enzymatic assay to the LC system is the elimination of the need for continuous infusion of the enzyme substrate into the MS source, which can increase the noise caused by overloading the MS detector, impairing the bioassay readout (de Boer et al., 2004; Kool et al., 2011; Yuan et al., 2020). It also allows for a time-controlled fraction transfer of the ^1^D effluent from the analytical column to the bioreactor. In this way, a sufficient number of fractions can be obtained from the chromatogram eluting from the ^1^D column which can facilitate the correlation of the ^1^D effluent fractions exhibiting inhibitory activity with specific regions in the chromatogram of the crude extract for the discovery of new potential enzyme inhibitors.

For the development of the 2D-LC-MS online platform for the AChE-cIMER inhibition assay, it was necessary to carry out a series of preliminary evaluations. The 2D-LC-MS online platform for the AChE-cIMER inhibition assay shown in Figure 3 is based on the same configuration used for comprehensive 2D-LC separations. Under these conditions compromises must be made between the ^1^D flow rate, modulation time, volume of the ^1^D effluent transferred to ^2^D (sample loop size), bioreactor length and residence time (D’Attoma et al., 2012). For a given ^1^D flow rate, it takes a certain amount of time to fill the sample loop installed in the interface. If the modulation time is longer than this filling time, some ^1^D effluent is lost to waste, and some of the components of the sample are not evaluated by the bioassay. The modulation time is also strongly related to the volume of the fraction of the ^1^D effluent transferred to the ^2^D. On the other hand, the ^2^D flow rate has to be just right enough to flush the sample in the loop toward the bioreactor, to perform the enzymatic reaction, and to re-condition the bioreactor before the next analysis all within one modulation period. Here again, compromises are made. For instance, a small ^2^D flow rate might be good for the enzymatic reaction, but it may require too much time to flush the sample plug from the sample loop, which would in turn require a long modulation time. In contrast, high ^2^D flow rates might be effective for flushing the sample out of the sample loop toward the bioreactor, but then may not allow enough time for the enzymatic reaction to occur. In summary, a shortest modulation time, a compatible sample loop size, and the highest ^2^D flow rate that can still allow the enzymatic reaction to be effectively monitored are desired (Sarrut et al., 2015; Stoll, 2017).

First, we evaluated the enzyme activity after the immobilization procedure using the system configuration described in Figure 1. The enzymatic activity after the immobilization procedure was characterized by the detection of the signal of the protonated choline molecular ion (*m/z* 104.0) corresponding to the enzymatic reaction product. Enzyme and substrate buffer conditions were chosen based on previous works develop by our group (Seidl et al., 2019). The syringe pump flow rate was set as 100 μl min^−1^ because it was the optimal recommended flow rate for the Gemini C18 analytical column that would be used in the final online method. Then, modulation time, volume of the ^1^D effluent transferred to the bioreactor (sample loop size), bioreactor length and residence time were adjusted accordingly. It was necessary to prepare a longer bioreactor (100 cm) containing a higher amount of immobilized enzyme to provide sufficient residence time between the enzyme and the substrate when using high ^2^D flow rates. The enzymatic reaction proved to be fast and reproducible for all the ^2^D flow rates tested. We observed that among the sample loop tested, with any size bigger than 60 μl the amount of methanol transferred (along with the sample) caused a decrease in enzyme activity and even death of the bioreactor. However, sample loop sizes smaller than 60 μl (40 and 20 μl) required very fast modulation times (24 and 12 s respectively) which affected the performance of the enzymatic reaction. Therefore, for the final method we used a modulation time of 36 s, a 60 μL sample loop, and ^2^D flow rate of 600 μl min^−1^. Using these optimal conditions, we obtained K_M,app_ (64.39 ± 6.58 μM). Normally, in enzyme screening assays, it is not known what type of inhibitors will be present and, therefore, it is important to set the substrate concentration close to K_M,app_ to balance the sensitivity of the assay and detect competitive, uncompetitive and non-competitive inhibitors (Copeland, 2016).

Next, using the system configuration described in Figure 2 we proceeded with the preliminary evaluation of the known acetylcholinesterase inhibitors galanthamine, tacrine and donepezil. In this configuration, substrate and inhibitors (or blank) solutions were delivered separately and combined through a T-piece placed before the switching valve to simulate what would happen in the final 2D-LC-MS online platform. The syringe pump represented ^1^D effluent flow eluting from the analytical column. Using this setup, it was possible to evaluate the effectiveness of the T connection in combining the inhibitor and substrate solutions, test the predetermined optimal conditions, and obtain the appropriate concentrations of inhibitors required to detect an appropriate enzyme inhibition.

Lastly, we evaluated the complete 2D-LC-MS online platform for the AChE-cIMER inhibition assay. For the HPLC portion of the 2D-LC/MS online platform, we selected the Gemini C18 (150 mm × 1 mm, 5 μm, 110 Å, Phenomenex, Torrance, United States) as the analytical column. Reverse-phase liquid chromatography in gradient mode is still the most widely used technique to separate complex NP-derived mixtures containing components with a wide range of capacity factors with high resolving power. In previous works, the enzyme activity was strongly impaired in the presence of the organic modifier even when the MeOH concentration was kept as low as 25% (Ingkaninan et al., 2000b; Yuan et al., 2020). Here, due to a combination of dilution effects inherent to the system configuration (Figure 3) the enzyme activity was only slightly affected when the MeOH concentration in the ^1^D effluent reached 100% (Figure 6 Panel 1A-D). However, the enzyme activity was recovered prior to the next injection after flushing with 15 mM ammonium acetate solution at pH 8.

Each known inhibitor was first evaluated individually followed by an ion suppression verification experiment to check if the inhibitor when eluting from the HPLC column influenced the ionization of the choline ion (*m/z* 104). As the MS detection of the inhibition assay was based on the visualization of a decrease in the intensity of the ion signal corresponding to the enzymatic reaction product (m/z 104), it was important to verify the occurrence of the signal suppression to prove that the decrease in the signal was indeed due to true enzyme inhibition. Once inhibition was confirmed, a solution containing the three standard inhibitors was analyzed and the same inhibitory profile was obtained, confirming the platform’s ability to screen for AChE inhibitors in a mixture.

To demonstrate the applicability of the complete 2D-LC-MS online platform for the AChE-cIMER inhibition assay for the analysis of complex samples such as those derived from NPs, a sample of an ethanolic extract obtained from *H. calyptratum* bulbs was used. This species was particularly chosen because it belongs to the Amaryllidaceae family, which is known to have the well-known AChE inhibitor galanthamine in its chemical composition. Previous studies (de Andrade et al., 2014) reported the presence of this alkaloid in this species and, therefore, it was an excellent sample for a proof of concept study. The difference in the intensity of inhibition observed between standard galanthamine and galanthamine present in the extract can be explained due to the lower concentration of galanthamine within the extract. Therefore, by using the complete 2D-LC-MS online platform for the AChE-cIMER inhibition assay proposed here it was possible to 1) confirm the expected inhibition caused by galanthamine present in the extract 2) identify two other regions of inhibition not related to galanthamine that may indicate one or a set of compounds with apparently greater anticholinesterase activity within the extract. All of this was accomplished with a 40-min analysis, using only 10 μL of sample and without the need for any sample pretreatment.

In summary, we developed a 2D-LC-MS online platform for AChE-cIMER inhibition assay capable of screening for AChE inhibitors in complex matrices such as those derived from NPs without the need for any sample treatment. This platform has the advantage of using commercially available instrumentation and software, as well as a well-established type of reusable immobilized enzyme bioreactor. The addition of the switching valve as the system interface eliminated the need for constant infusion of the substrate solution in the MS, allowed the determination of K_M,app_ and the performance of inhibition assays under favorable conditions for the screening of complex samples containing different type of inhibitors. Taking advantage of a 2D-LC configuration in comprehensive mode, one can maintain the separation achieved in the first dimension and therefore facilitate the correlation of fractions of the ^1^D effluent exhibiting inhibitory activity with specific regions in the chromatogram of the crude extract. It is then possible to only target these regions to discover new potential AChE inhibitors, eliminating fractionation steps thus contributing to decrease the time involved in NPs bioprospecting.

## Data Availability

The raw data supporting the conclusion of this article will be made available by the authors, without undue reservation.
