# Supplementary figures and images for: A Comprehensive 2D-LC/MS Online Platform for Screening of Acetylcholinesterase Inhibitors
Source: Front Mol Biosci. 2022 Mar 16;9:868597. doi: 10.3389/fmolb.2022.868597 (PMC8967351; doi:10.3389/fmolb.2022.868597)

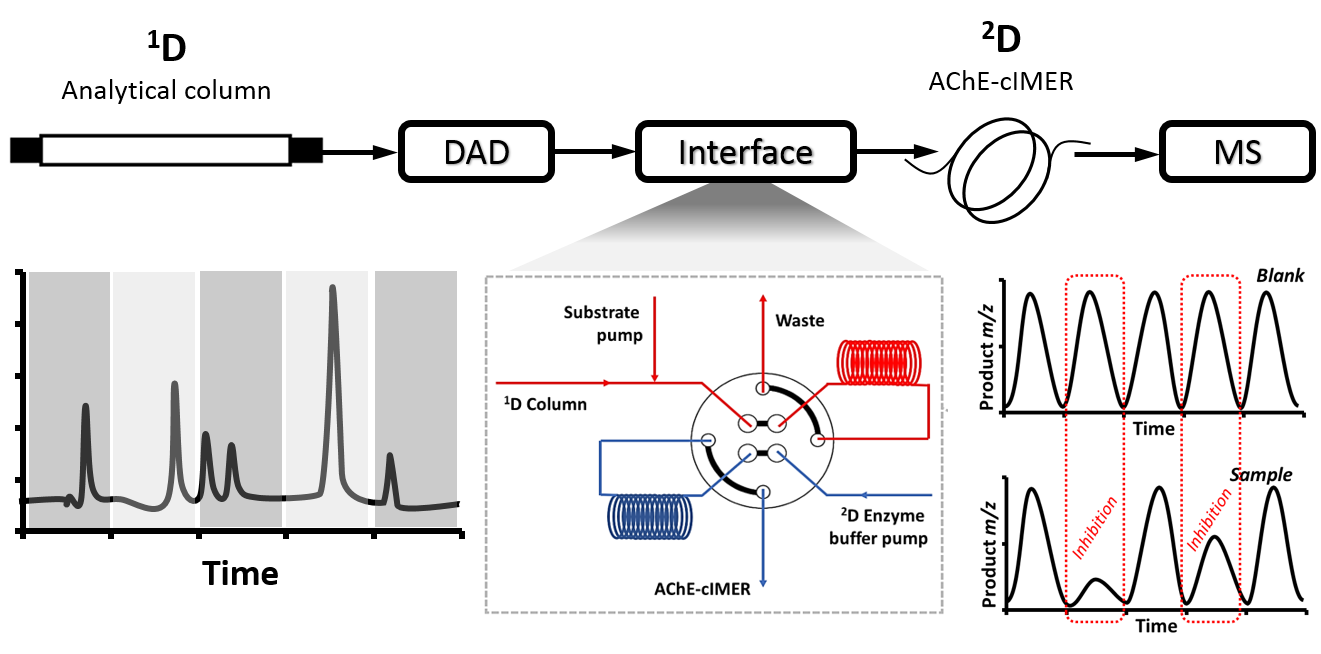

Supplement: Supplementary file 1 [file Image1.TIFF]

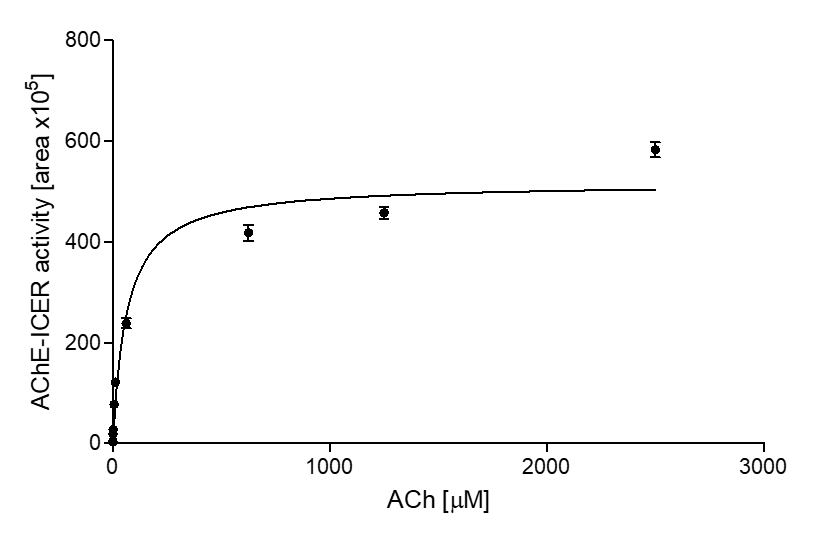

Supplement: Supplementary file 2 [file Image3.TIF]

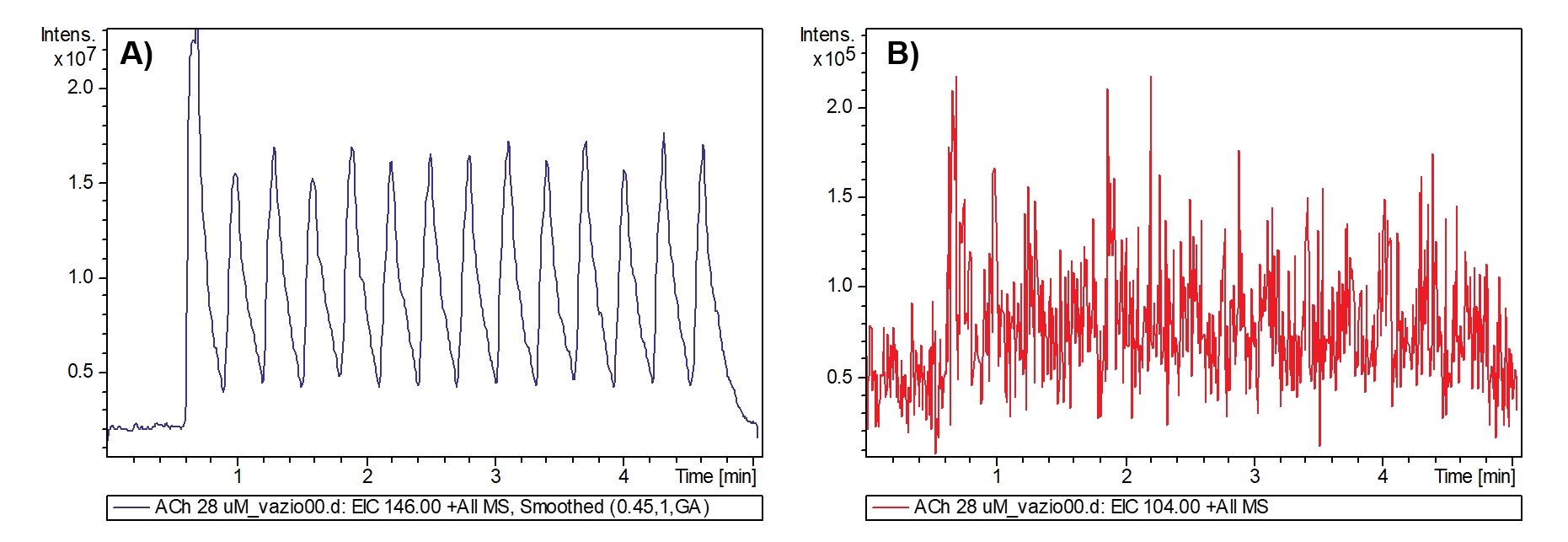

Supplement: Supplementary file 3 [file Image4.TIF]

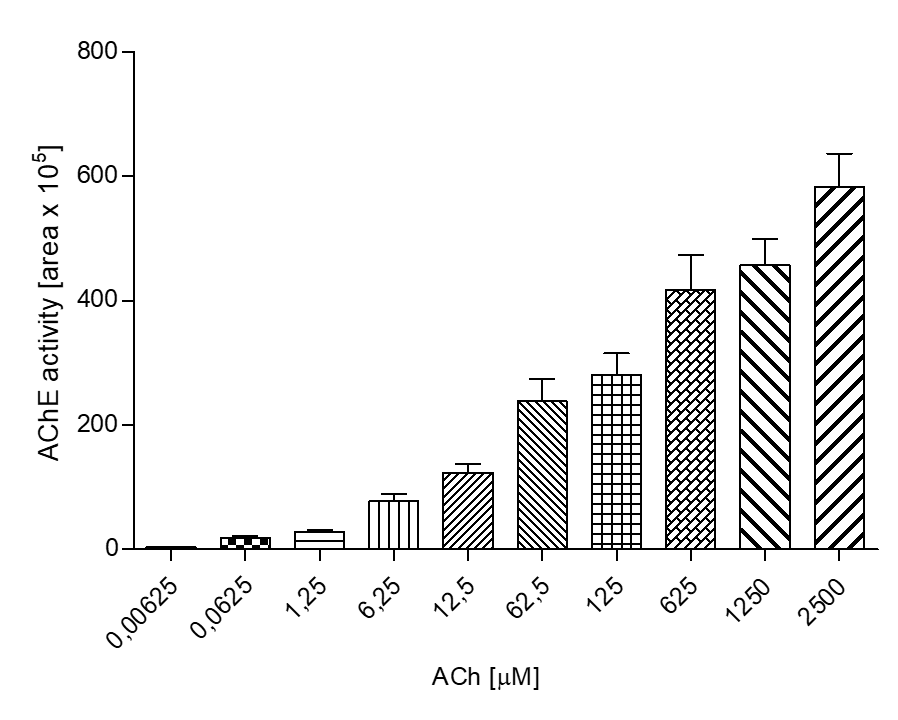

Supplement: Supplementary file 4 [file Image2.TIF]
