# Supplementary material for: A Comprehensive 2D-LC/MS Online Platform for Screening of Acetylcholinesterase Inhibitors
Source: Front Mol Biosci. 2022 Mar 16;9:868597. doi: 10.3389/fmolb.2022.868597 (PMC8967351; doi:10.3389/fmolb.2022.868597)
Supplement: Supplementary file 5 [file DataSheet1.docx]

**Supplemental Figures**

**A comprehensive 2D-LC/MS online platform for screening of acetylcholinesterase inhibitor**

Claudia Seidl^a^, Juliana Maria Lima^a^, Gabriel Mazzi Leme^b^, Ananda Ferreira Pires^a^, Dwight R. Stoll^c^, Carmen Lúcia Cardoso^a^*

*^a^Departamento de Química, Grupo de Cromatografia de Bioafinidade e Produtos Naturais, Faculdade de Filosofia, Ciências e Letras de Ribeirão Preto, Universidade de São Paulo, Ribeirão Preto, 14040-90, Brazil*

^b^*SEPARARE Núcleo de Pesquisa em Cromatografia, Departamento de Química, Universidade Federal de São Carlos, Caixa Postal 676, São Carlos 13565-905, Brazil*

*^c^Department of Chemistry, Gustavus Adolphus College, St. Peter, Minnesota, 56082, United States*

*Corresponding author: ccardoso@ffclrp.usp.br

**Supplemental Figure 1**

**S1A.** Verification of the AChE- cIMER catalytic activity after the immobilization procedure. Eight ACh sequential concentrations ranging from 2.5 to 6.25 x 10^-5^ mM prepared in 15 mM ammonium acetate solution pH 5 were infused into the system described in Figure 1 at 100 μL.min^-1^. Conditions: 600 μl.min^-1^ enzyme buffer pump flow rate, two 60 μL loop and 36 s as modulation time. Bars chart shows the resulting area corresponding to the protonated molecular ion of Choline (Ch, [M+H]^+^ *m*/*z* 104.0), product of acetylcholine (ACh) enzymatic hydrolysis by acetylcholinesterase (AChE). Results expressed as means +/- one standard deviation from 3 replicates.

From this graph it is possible to observe that the immobilization procedure did not affect the enzymatic activity. Moreover, the amount of enzyme used (50 U.mL^-1^) proved to be enough to deliver a reliable, reproducible, and detectable signal for the protonated molecular ion of choline (*m/z* 104.0), the product of the enzymatic reaction (Figure 4), even when the substrate concentration was low as 6.25x10^-5^ μM.

**S1B**. The Michaelis–Menten saturation curve for the AChE-cIMER. Eight ACh sequential concentrations ranging from 2.5 to 6.25 x 10^-5^ μM prepared in 15 mM ammonium acetate solution pH 5 were infused into the system described in Figure 1 at 100 μL.min^-1^. Conditions: 600 μl.min^-1^ enzyme buffer pump flow rate, two 60 μL loop and 36 s as modulation time. Results expressed as means +/- one standard deviation from 3 replicates.

From this experiment, the apparent Michealis-Mentem constant K_M,app_ (64.39 ± 6.58 μM) from the Michaelis–Menten saturation curve for the AChE-cIMER. AChE- cIMER inhibition screening assays were carried out at substrate concentration close to K_M,app_ value of 70 µM.

**Supplemental Figure 2**

**S2.** Evaluation of spontaneous hydrolysis of the substrate — Panel A showing the signal corresponding to the substrate of the enzyme reaction ion, acetylcholine (ACh, [M+H]^+^*m/z* 146.0). Panel B showing the absence of a detectible signal corresponding to the product of the enzyme reaction ion, choline (Ch, [M+H]^+^ *m*/*z* 104.0) when the substrate solution was infused was infused through the cIMER-blank.

Spontaneous hydrolysis of the substrate was barely detectable — only noise signal corresponding to the Ch product ion (*m/z* 104.0) was detected when the substrate solution was infused through the cIMER-blank.
